# Pulmonary fibrosis in patients with COVID-19: A retrospective study

**DOI:** 10.3389/fcimb.2022.1013526

**Published:** 2022-12-21

**Authors:** Fanglin Li, Jiayi Deng, Yongqiang Song, Chenfang Wu, Bo Yu, Guyi Wang, Jinxiu Li, Yanjun Zhong, Fang Liang

**Affiliations:** ^1^ Department of Hematology and Key Laboratory of Non-resolving Inflammation and Cancer of Hunan Province, The Third Xiangya Hospital, Central South University, Changsha, Hunan, China; ^2^ Critical Care Medicine, The Second Xiangya Hospital, Central South University, Changsha, Hunan, China; ^3^ Critical Care Medicine, Renmin Hospital of Yiyang, Yiyang, Hunan, China

**Keywords:** COVID-19, pulmonary fibrosis, inflammation, coagulopathy, fibrinolysis

## Abstract

**Background:**

Coronavirus disease 2019 (COVID-19) pandemic has caused substantial threats to people’s physical health and lives, claiming the lives of over 6 million people worldwide. Although the mortality rate of COVID-19 is very low, many survivors may have different degrees and various sequelae. Previous studies have shown that pulmonary fibrosis (PF) were common on discharged COVID-19 patients, and PF itself is a poor prognostic factor.

**Methods:**

227 COVID-19 hospitalized patients’ clinical and laboratory data from the first 15 days following admission were collected in this retrospective study. Groups were based on with or without PF of COVID-19. Categorical variables were compared with the chi-square test or Fisher’s exact test. Continuous variables were tested by Wilcoxon rank-sum test for the non-normal distribution. Spearman correlations were used to assess the correlations between PF with clinic parameters of multiple time points. Univariate and multivariate logistic regression were used to analyze for risk factors of COVID-19 patients with pulmonary fibrosis.

**Results:**

Sixty cases of COVID-19 patients were diagnosed with PF. Compared with 167 non-PF patients, those with PF were older and had higher proportions of fever, shortness of breath, hemoptysis, abdominal pain, hypertension, cardiovascular, diabetes, high flow nasal cannula (HFNC), severe disease, and virus shedding duration. Furthermore, the correlation analysis between PF and clinic parameters showed that PF were positively related to the C-reactive protein (CRP) and erythrocyte sedimentation rate (ESR), and negatively correlated with hemoglobin (HGB) and albumin (ALB) at all time points in the first 15 days after admission. Moreover, We found that PF were significantly correlated with coagulation indexes prothrombin time (PT), activated partial thromboplastin time (APTT), fibrinogen (Fib) and fibrinolysis index D-Dimer at some phases. In addition, Univariate logistic regression analyses showed that age, fever, shortness of breath, hemoptysis, hypertension, cardiovascular, diabetes, HFNC, severe disease were the risk factors of COVID-19 patients with PF. However, multivariate logistic regression showed that age was the risk factor of COVID-19 patients with PF.

**Conclusion:**

Combining various factors, advanced age is an independent risk factor of COVID-19 patients with PF. PF was significantly related with clinic parameter of inflammation/coagulopathy/fibrinolysis.

## 1 Introduction

Globally, the emergence of COVID-19 presents substantial healthcare, societal, and economic issues. Despite the fact that the majority of patients survived from severe acute respiratory syndrome coronavirus 2 (SARS-CoV-2) infection, it is usual for survivors to suffer a variety of complications after recovery (Ruggiero et al., 2022). Some survivors get pulmonary fibrosis (PF). PF is a pathological consequence of interstitial lung diseases that is marked by failure to repair the damaged alveolar epithelium, persistence of fibroblasts and excessive deposition of extracellular matrix (Umemura et al., 2021). Moreover, PF is not rare, following a severe COVID-19 infection, especially in mechanically ventilated patients (Sun et al., 2020; McGroder et al., 2021). PF is thought to be one of the most serious complications in patients recovered from COVID-19.

Previous studies suggested that most post-discharge COVID-19 patients have PF sequelae (Sun et al., 2020; Li et al., 2021). COVID-19 patients with pre-existing idiopathic pulmonary fibrosis (IPF) have been thought to have worse outcomes (Naqvi et al., 2021). IPF as a type of PF, is demonstrated to be associated with poor prognosis in non-COVID-19 patients (Lederer and Martinez, 2018). In addition to IPF, pneumonia, particularly severe pneumonia, is another significant cause of PF. SARS-CoV-2 infection can result in cytokine storm, which further causes ARDS, multiple organ dysfunction and even death. However, population based study from the United States suggested that the incidence of post-inflammatory fibrosis is 11 times higher than that of IPF (Raghu et al., 2006), and PF is a well-recognized sequela of ARDS (Villar et al., 2019). SARS-CoV-2, a respiratory virus, as its name indicates, the lungs are the primary site of infection and injury, and lead to pneumonia. Meanwhile, there is no clearly proven drugs against PF, which reduces the quality of life of patients and complicates the condition of COVID-19 and non-COVID-19 patients (Ojo et al., 2020). Therefore, finding prospective factors that can predict COVID-19 associated PF is extremely crucial for a better prognosis.

The lung is the main target of SARS-CoV-2, resulting in adverse respiratory function consequences. Such as lung inflammatory response induced by viral infection and bacterial infection after secondary viral infection, as well as endothelial dysfunction and microvascular injury. These can contribute to persistent lung damage, including the recovery period after discharge, and may eventually lead to PF (John et al., 2021). The pathogenesis of PF in COVID-19 have been discussed in several studies (John et al., 2021; Susanto et al., 2021; Xiang et al., 2022) and alveolar epithelial cell injury during lung infection with SARS-CoV-2 are reported to be the major cause for the occurrence of PF since they lead to infiltration of fibroblasts and inflammatory cells, resulting in the release of pro-fibrotic factors (Martinez et al., 2017). Meanwhile, 83% of type II alveolar cells express angiotensin-converting enzyme 2 (ACE2), which is the primary receptor for SARS-CoV-2 and may partly explain alveolar epithelial cell injury (Umemura et al., 2021; Bui et al., 2021). Furthermore, the use of mechanical ventilation in COVID-19 patients might exacerbate alveolar cell damage. Following alveolar cell injury, transforming growth factor beta (TGF-β) releases to aid in lung healing. PF is frequently brought on by the excessive activation of the TGF-β pathway when suffered from viral infection (Umemura et al., 2021). However, clinical risk factors associated with PF after COVID-19 infection remain not very clear. Our study’s primary objective was to better understand the risk factors for developing PF following COVID-19 infection.

In this retrospective investigation of COVID-19 patients, the clinical, and laboratory data of 227 enrolled patients were evaluated.

## 2 Methods

### 2.1 Design and participants

This retrospective study enrolled 227 confirmed COVID-19 patients, who hospitalized in the Public Health Treatment Center, Changsha, China, as of March 26^th^, 2020 and had chest computed tomography (CT) within 1 year after discharge [29 (24, 31) days] (**Table 1**). The patients accompanied with a history of PF were excluded. The patients did not receive any post-discharge anti-inflammatory treatment for pro-fibrotic during follow-up. COVID-19 cases were classified into two groups depending on chest CT within 1 year after discharge. Based on the CT scoring system for fibrosis, the patients with the scoring reach 1 were classified as the PF group as reported in the previous study: 1) No fibrosis, score 0; 2) Interlobular septal thickening, no discrete honeycombing, score 1; 3) Honeycombing (with or without septal thickening) involving <25% of the lobe, score 2; 4) Honeycombing involving 25-49% of the lobe, score 3; 5) Honeycombing involving 50-75% of the lobe, score 4; 6) Honeycombing involving >75% of the lobe, score 5 (Yamaguchi et al., 2018). We categorized the study period into five separate phases to better describe, with three days for each phase, based on the surrounding days when there were no statistically significant variations in the laboratory results: T0 to T4. This study was subjected to the approval by the institutional ethics board of the Second Xiangya hospital.

**Table 1:** Baseline characteristics of patients with or without pulmonary fibrosis.

	PF group(n = 60)	Non-PF group(n = 167)	All patients(n = 227)	*P* value
Gender (male/female)	22 (36.67)	85 (50.90)	107 (47.14)	0.058
Age, y, M (IQR)	64 (47, 67)	40 (30.5, 51.5)	45 (34, 59)	< 0.001***
Drinking (n, %)	2 (3.33)	6 (3.59)	8 (3.52)	0.246
Smoking (n, %)	2 (3.33)	15 (8.98)	17 (7.49)	0.251
Time from discharge to CT reexamination, d, M (IQR)	29 (25.5, 32)	29 (24, 31)	29 (24, 31)	0.563
Symptoms				
Fever (n, %)	52 (86.67)	114 (58.26)	166 (73.13)	0.006**
Fatigue (n, %)	31 (51.67)	69 (41.32)	100 (44.05)	0.166
Cough (n, %)	49 (81.67)	132 (79.04)	181 (79.74)	0.664
Expectoration (n, %)	29 (48.33)	72 (43.11)	101 (44.49)	0.485
Shortness of breath (n, %)	34 (56.67)	40 (23.95)	74 (32.60)	< 0.001***
Hemoptysis (n, %)	4 (6.67)	1 (0.60)	5 (2.20)	0.018*
Diarrhea (n, %)	16 (26.67)	34 (20.36)	50 (22.03)	0.312
Abdominal pain (n, %)	4 (6.67)	28 (16.77)	32 (14.10)	0.038*
Myalgia (n, %)	8 (13.33)	15 (8.98)	23 (10.13)	0.338
Headache (n, %)	11 (18.33)	15 (8.98)	26 (11.45)	0.051
Chills (n, %)	7 (11.67)	20 (11.98)	27 (11.89)	0.949
Soreness (n, %)	8 (13.33)	15 (8.98)	23 (10.13)	0.338
Nausea (n, %)	9 (15.00)	20 (11.98)	29 (12.78)	0.547
Vomiting (n, %)	5 (8.33)	20 (11.98)	25 (11.01)	0.439
Comorbidities				
Hypertension (n, %)	19 (31.67)	16 (9.58)	35 (15.42)	< 0.001***
Cardiovascular (n, %)	6 (10.00)	2 (1.20)	8 (3.52)	0.005**
Diabetes (n, %)	10 (16.67)	5 (2.30)	15 (6.61)	< 0.001***
Treatment				
Glucocorticoid (n, %)	34 (56.67)	35 (20.96)	69 (30.40)	**< 0.001**
Immunoglobulin (n, %)	33 (55.00)	32 (19.16)	65 (28.62)	**< 0.001**
Outcomes				
HFNC (n, %)	9 (15.00)	2 (1.20)	11 (4.85)	< 0.001***
Noninvasive ventilator (n, %)	2 (3.33)	1 (0.60)	3 (1.32)	0.171
Invasive ventilator (n, %)	1 (1.67)	0 (0.00)	1 (0.44)	0.264
Severe disease	21 (35.00)	18 (10.78)	39 (17.18)	< 0.001***
Virus shedding duration	20 (15, 29)	17 (13, 24)	18 (13, 24)	0.042*

* means a P < 0.05 vs Non-PF, ** means a P < 0.01 vs Non-PF, *** means a P < 0.001 vs Non-PF, PF, pulmonary fibrosis, M, median, HFNC, high flow nasal cannula.

Bold values/numbers means p<0.05 and statistics significant.

### 2.2 Data collection

The data were retrospectively collected from electronic medical records. The following data were collected: demographic, symptoms, underlying diseases, outcomes, laboratory parameters within first 15 days after admission, and chest CT within 1 year after discharge.

### 2.3 Statistical analysis

In this study, we used median with IQR and frequencies to describe continuous and categorical variables, respectively. The differences between PF and non-PF groups were compared using Wilcoxon rank-sum test for the non-normal distribution for continuous variables and Fisher’s exact test or chi-square test for categorical variables. Logistic regression analyses were used to determine the association between variables and PF. Spearman correlations were used to assess the correlations between PF scores with clinic parameters of multiple time points. All above analyses were performed using SPSS 26.0 and R language.

## 3 Results

This study included in 227 confirmed COVID-19 patients, among which 60 cases were diagnosed as PF of which 59 cases were 1 point. Fever [52 (86.67%)], cough [49 (81.67%)], shortness of breath [34 (56.67%)], fatigue [31 (51.67%)] and expectoration [29 (48.33%)] were the most common symptoms in COVID-19 patients with PF. Patients of PF group were significantly older than non-PF patients [64 (47, 67) vs 40 (30.5, 51.5), *P* < 0.001]. What’s more, PF patients were more likely to have experienced shortness of breath, fever, hemoptysis and abdominal pain than control patients symptoms [34 (56.67%) vs 40 (23.95%), *P* < 0.001; 52 (86.67%) vs 114 (58.26%), *P* = 0.006; 4 (6.67%) vs 1 (0.6%), *P* = 0.018; 4 (6.67%) vs 28 (16.77%), *P* = 0.038]. Regarding concomitant diseases, PF patients have greater percentages of hypertension, cardiovascular disease and diabetes when compared to patients in the non-PF group (31.67% vs 9.58%, *P* < 0.001; 10% vs 1.2%, *P* = 0.005 and 16.67% vs 2.3%, *P* < 0.001). In addition, PF patients were more likely to use glucocorticoid (56.67% vs 20.96%, *P* < 0.001), and immunoglobulin (55.00% vs 19.16%, *P* < 0.001). The most striking result to emerge from the table is that PF group had higher proportions of severe disease (35% vs 10.78%, *P* < 0.001) and high flow nasal cannula (HFNC) (15% vs 1.2%, *P* < 0.001), and a longer virus shedding duration [20 (15, 29) vs 17 (13, 24) days, *P* = 0.042] (**Table 1**).

Our data also demonstrated the dynamic process during the study period, and it indicated the differences in critical laboratory parameters between the PF and non-PF groups. The inflammatory biomarkers in PF patients were significantly higher than that of non-PF group. There was an increase in the white blood cell (WBC) count from T0 to T4. Procalcitonin (PCT) at T0 was the highest, while lymphocytes (Lys) at T1 was the lowest. Erythrocyte sedimentation rate (ESR) had an increasing tendency from T0 to T3, and C-reactive protein (CRP) decreased from T0 to T4 while T1 had an increasing tendency. In terms of coagulation parameters, D-dimer had an increasing tendency from T0 to T4, while T2 was significantly decreased and platelet count increased from T0 to T3. Prothrombin time (PT) and activated partial thromboplastin time (APTT) decreased from T0 to T3. Fibrin degradation product (FDP) decreased throughout all time points (**Table 2**). Compared with the non-PF group, PF patients had a significantly higher levels of CRP and ESR, whereas hemoglobin (HGB) (from T0 to T4), Lys (from T0 to T3) and APTT (T2, T3) were obviously lower in PF patients than those of non-PF patients. PCT (T0), PT (T2), FDP (T0) and D-Dimer (from T1 to T4) were significantly higher in PF patients. With regard to the liver function parameters, the levels of alanine aminotranspherase (ALT) (T1) and aspartate aminotransferase (AST) (from T0 to T2), total bilirubin (TBil) (T2) were obviously higher in patients with PF, while the albumin (ALB) were significantly lower during the study period. Considering kidney function, serum urea nitrogen (BUN) was significantly higher in PF patients at T1 (**Table 2**).

**Table 2:** Laboratory findings of patients with or without pulmonary fibrosis at different time points after admission.

	T0 (D0-D2)		T1(D3-D5)		T2 (D6-D8)		T3 (D9-D11)		T4 (D12-D14)	
	PF	Non-PF	PF	Non-PF	PF	Non-PF	PF	Non-PF	PF	Non-PF
	Median (IQR)	Median (IQR)	Median (IQR)	Median (IQR)	Median (IQR)	Median (IQR)	Median (IQR)	Median (IQR)	Median (IQR)	Median (IQR)
WBC	4.52 (3.35, 5.62)	4.65 (3.50, 5.87)	5.6 (4.05, 8.00)	5.2 (4.03, 6.99)	6.39 (4.85, 8.50)	5.65 (4.46, 7.41)	6.8 (5.43, 9.05)	6.16 (5.11, 8.40)	6.92 (6.19, 9.20)	6.62 (5.35, 8.90)
HGB	125 (116, 136)*	131 (121, 143)	124 (113, 131.5)**	133.5 (121, 145)	122 (112, 131.5)***	131.25 (119, 144)	118 (109, 125)***	130 (114, 140)	118 (107.5, 130)**	130 (117, 140.25)
PLT	162 (131, 202)	173 (139, 232)	215 (138, 274.5)	206.5 (151, 262)	249 (181.25, 309.5)	228 (178.5, 291)	280.75 (208, 314.25)	241.5 (192.5, 302)	268.5 (210.75, 338)	249 (191, 308)
Lys	0.90 (0.63, 1.12)***	1.29 (0.95, 1.84)	0.8096 (0.62, 1.26)***	1.33 (0.98, 1.75)	0.87 (0.58, 1.47)***	1.34 (0.99, 1.77)	1.28 (0.71, 1.63)**	1.47 (1.1, 1.86)	1.28 (1.02, 1.89)	1.54 (1.20, 1.87)
CRP	32.76 (16.39, 54.67)***	8.86 (2.90, 21.45)	34.2 (15.87, 45.71)***	10 (4, 21.75)	11.8 (6.2, 46.19)**	6.62 (2.8, 12.23)	6.95 (3.23, 13.66)**	3.5 (1.7, 7.6)	5.2 (2.5, 11.67)*	2.92 (1.15, 7.65)
PCT	0.04 (0, 0.08)***	0.00 (0.00, 0.04)	0.00 (0.00, 0.07)	0.00 (0.00, 0.04)	0 (0, 0.012)	0 (0, 0.04)	0 (0, 0)	0 (0, 0.031)	0 (0, 0)	0 (0, 0)
ESR	58 (41, 79)***	28 (16.25, 57.50)	75.5 (48, 98)***	43 (26.25, 66.75)	80 (63.25, 101.75)***	51.5 (29, 79.25)	81 (53, 95)**	56 (29, 78)	72 (36.25, 91)*	40.5 (15.75, 81.5)
PT	12.1 (11.2, 12.68)	11.8 (11.1, 12.38)	11.53 (10.83, 12.28)	11.4 (10.8, 12)	11.1 (10.6, 12.05)*	10.8 (10.4, 11.2)	10.8 (10.4, 11.5)	10.7 (10.2, 11.2)	10.85 (10.3, 11.7)	10.8 (10.5, 11.4)
APTT	32.1 (29.45, 34.2)	32.65 (30.8, 35.4)	31.53 (27.83, 33.95)	31.95 (30.28, 34.35)	29.9 (27.15, 32.5)*	31.7 (29.8, 34.1)	29.1 (25.7, 31.38)*	30.9 (28.4, 34)	29.75 (25.2, 34.3)	31 (28.8, 34)
FDP	3.97 (3.44, 4.68)***	3.41 (2.73, 3.97)	3.86 (3.62, 4.5)	3.62 (3, 4.5)	3.74 (2.90, 4.08)	3.52 (2.93, 4.08)	3.55 (2.82, 3.81)	3.17 (2.73, 3.62)	3.29 (2.64, 3.58)	2.94 (2.49, 3.36)
D-dimer	0.26 (0.14, 0.77)	0.25 (0.13, 0.49)	0.35 (0.14, 0.64)**	0.21 (0.09, 0.39)	0.27 (0.17, 1.37)**	0.18 (0.10, 0.36)	0.40 (0.14, 1.20)**	0.15 (0.08, 0.47)	0.55 (0.19, 2.99)**	0.18 (0.10, 0.53)
ALT	19.91 (15.8, 29.13)	18.35 (13.60, 26.41)	20.35 (15.76, 28.95)*	17.8 (13.44, 24.7)	24.05 (17.13, 37.05)	19.3 (15, 35.2)	26.4 (17.7, 48.3)	27.2 (15.75, 42.5)	34.3 (19.15, 50.7)	26.8 (17.55, 53.3)
AST	26.9 (22.52, 38.84)***	23.38 (18.85, 28.54)	25.95 (20.39, 32.06)**	21.4 (17.1, 27.8)	25.36 (18.95, 37.35)*	22.05 (17.4, 29.2)	27.46 (19.16, 32.4)	23 (17.4, 30.65)	26.2 (19.8, 39.43)	25.2 (18.35, 32.98)
TBil	11.91 (9.09, 16.32)	10.61 (8.24, 15.32)	14.05 (8.73, 21.3)	13.7 (9.7, 20.11)	11.4 (8.08, 14.61)*	9.8 (7.4175, 13)	10.3 (7.7, 12.7)	9 (6.75, 12.55)	9.7 (7.25, 14.44)	9.5 (7.48, 12.65)
Alb	35.615 (33.87, 38.48)***	39.12 (36.27, 42.09)	34.27 (31.33, 38.6)***	38.8 (36.3, 41.7)	33.85 (31.13, 37.9)***	39 (35.97, 41.8)	34.6 (30.7, 37.7)***	39.7 (36.6, 42.65)	36.5 (31.45, 39.9)***	40.9 (36.75, 44.6)
Cr	50.05 (40.75, 62.59)	51.13 (40.32, 63.84)	54.9 (43.1, 61.23)	55.56 (43.08, 70.53)	51.15 (41.58, 64.95)	53.2 (42.23, 68.6)	53 (45.4, 62.6)	56.8 (46.6, 72.05)	54.8 (44.91, 66.18)	55.6 (46.25, 69.15)
BUN	4.65 (3.18, 6.73)	4.30 (3.41, 4.89)	5.42 (3.77, 7.38)*	4.5 (3.76, 5.51)	5.75 (3.7, 7.39)	4.8 (3.98, 5.9)	6.03 (4.35, 7.26)	5.09 (4.28, 6.4)	5.96 (4.74, 7.43)	5.2 (4.5, 6.3)

* means a P < 0.05 vs Non-PF, ** means a P < 0.01 vs Non-PF, *** means a P < 0.001 vs Non-PF, PF, pulmonary fibrosis, SE, standard error, WBC, white blood cell count, HGB, hemoglobin, PLT, platelet, CRP, C-reactive protein, PCT, procalcitonin, ESR, erythrocyte sedimentation rate, PT, prothrombin time, APTT, activated partial thromboplastin time, FDR, fibrinogen, ALT, alanine aminotranspherase, AST, aspartate aminotransferase, TBil, total bilirubin, Alb, albumin, Cr, serum creatinine, BUN, urea nitrogen.

**Figure 1:**
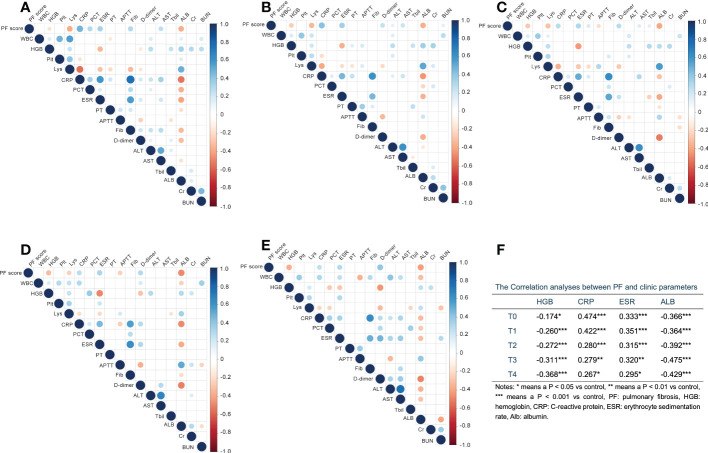
The correlation analysis between pulmonary fibrosis (PF) score and clinic parameters at different time points with first 15 days after admission. **(A–E)** Digits represents Pearson correlation coefficients, filled color indicates significant correlation, blue represents positive correlation, red represents negative correlation, and shade of color represents strength of correlation. A for T0, B for T1, C for T2, D for T3, E for T4. **(F)** The correlation indexes for parameters which significantly associated with PF at all time points. WBC, white blood cell; HGB, hemoglobin; Plt, platelet; Lys, lymphocytes; CRP, C-reactive protein; PCT, procalcitonin; ESR, erythrocyte sedimentation rate; PT, prothrombin time; APTT, activated partial thromboplastin time; Fib, fibrinogen; ALT, alanine aminotranspherase; AST, aspartate aminotransferase; TBil, total bilirubin; Alb, albumin; Cr, serum creatinine; BUN, urea nitrogen. *p < 0.05, **p < 0.01, ***p < 0.001.

To further understand the development and relevant risk factors of PF in patients with COVID-19, we then analyzed the association between PF and clinic indicators. As shown in **Figure 1**, HGB, CRP, ESR and ALB were significantly associated with PF score across all time points, with CRP and ESR positively, while HGB and ALB negatively. Lys from T0 to T3, and APTT (T2&T3) were significantly negatively related with PF score. PCT at T0, PT at T2, fibrinogen (Fib) at T0, D-dimer at T1 to T4, ALT at T1, AST at T0 to T2, TBil at T2 and BUN at T1 were significantly positively associated with PF score (**Figure 1**).

In addition, we used univariate and multivariate logistic regression analyses for risk factors of PF in patients with COVID-19. As illustrated, age [OR = 1.092 (1.064, 1.121), *P* < 0.001], fever [OR = 3.022 (1.341, 6.810), *P* = 0.008], shortness of breath [OR = 4.152 (2.229, 7.734), *P* = 0.008], hemoptysis [OR = 11.857 (1.298, 108.318), *P* = 0.028], hypertension [OR = 4.373 (2.067, 9.252), *P* < 0.001], cardiovascular [OR = 9.167 (1.797, 46.764), *P* = 0.008], diabetes [OR = 6.480 (2.116, 19.848), *P* = 0.001], Glucocorticoid [OR = 4.759 (2.535, 8.934), *P* < 0.001], Immunoglobulin [OR = 4.963 (2.629, 9.369), *P* < 0.001], HFNC [OR = 14.559 (3.047, 69.562), *P* = 0.001], severe disease [OR = 4.457 (2.166, 9.171), *P* < 0.001], HGB [OR = 0.975 (0.955, 0.995), *P* = 0.013], CRP [OR = 1.050 (1.033, 1.068), *P* < 0.001], ESR [OR = 1.024 (1.013, 1.036), *P* < 0.001] and ALB [OR = 0.801 (0.734, 0.874), *P* < 0.001] are all risk factors for PF in univariate regression analyses. Age was an independent risk factor for PF when the above factors were combined, and multivariate regression analysis was used [OR = 1.047 (1.010, 1.085), *P* = 0.012] (**Table 3**).

**Table 3:** Univariate and multivariate logistic regression analyses for risk factors at baseline of COVID-19 patients with pulmonary fibrosis.

	Univariate		Multivariate	
	OR (CI%)	*P* value	OR (CI%)	*P* value
Age	1.092 (1.064, 1.121)	**< 0.001**	1.047 (1.010, 1.085)	**0.012**
Fever	3.022 (1.341, 6.810)	**0.008**	NA	0.567
Shortness of breath	4.152 (2.229, 7.734)	**< 0.001**	NA	0.151
Hemoptysis	11.857 (1.298, 108.318)	**0.028**	NA	0.999
Hypertension	4.373 (2.067, 9.252)	**< 0.001**	NA	0.521
Cardiovascular	9.167 (1.797, 46.764)	**0.008**	NA	0.707
Diabetes	6.480 (2.116, 19.848)	**0.001**	NA	0.239
Glucocorticoid	4.759 (2.535, 8.934)	**< 0.001**	NA	0.745
Immunoglobulin	4.963 (2.629, 9.369)	**< 0.001**	NA	0.297
HFNC	14.559 (3.047, 69.562)	**0.001**	NA	0.441
Severe disease	4.457 (2.166, 9.171)	**< 0.001**	NA	0.999
HGB	0.975 (0.955, 0.995)	**0.013**	NA	0.860
CRP	1.050 (1.033, 1.068)	**< 0.001**	NA	0.268
ESR	1.024 (1.013, 1.036)	**< 0.001**	NA	0.539
ALB	0.801 (0.734, 0.874)	**< 0.001**	NA	0.094

HFNC, high flow nasal cannula.

Bold values/numbers means p<0.05 and statistics significant.

## 4 Discussion

This study indicated that age is an independent risk factor for PF. Likewise, our study demonstrated a potential association between PF and several significant indicators of inflammation, coagulopathy and organ function, which is probably relevant for the development, prognosis and sequelae of COVID-19.

We contrasted the PF group’s demographic characteristics, clinical symptoms, laboratory results, and comorbidities with those of the non-PF group. One of the findings is that PF was more likely to appear in the older patients. COVID-19 patients in particular were more likely to have PF together with hypertension, cardiovascular disease, or diabetes. The most common underlying diseases were hypertension, cardiovascular disease, and diabetes. The most frequent symptoms were cough and fever, according to prior investigations (Martinez et al., 2017; Zhou et al., 2020; Li et al., 2021).

In this study, age, fever, hemoptysis, shortness of breath, hypertension, cardiovascular, diabetes, glucocorticoid, immunoglobulin, HFNC, disease severity, HGB, CRP, ESR and ALB were risk factors for PF, which was in according with previous studies. It is worth mentioning that, combined with multiple clinical indicators, age is an independent risk factor for PF. One study included in 289 patients with COVID-19 indicated a significant association between COVID-19 severity and susceptibility of PF. PF was more likely to occur in patients with advanced age, fever, severe/critical disease, pre-existing disease and a longer virus shedding duration (Li et al., 2021). Another retrospective cohort study included in 191 COVID-19 patients also indicated older age was a risk factor for death (Zhou et al., 2020). Meanwhile, a study that used an observational cohort study also showed that the fibrotic patients were older compared to non-fibrotic patients. In keeping with our findings, the study also demonstrated that the progression of post COVID-19 PF is significantly influenced by comorbidities such diabetes, hypertension, and cardiovascular disease (Huang et al., 2021). Another study pointed out that PF was related with advanced age, and it was more prevalent in the persistent and chronic phases of COVID-19, while it may be not evident in the early stage, due to the time required for extracellular matrix deposition (Dsouza et al., 2022). In contrast, a recent study has also shown that the increased incidence of COVID-19 sequelae is age-independent (Daugherty et al., 2021). Moreover, it was reported that the major risk factors for severe COVID-19 are shared with PF (George et al., 2020). Comparable to what was said in a previous research, COVID-19 patients with pre-existing PF have been thought to have worse outcomes (Naqvi et al., 2021), which also proved by an international multicenter study (Drake et al., 2020). Most recently, a study reported that chronic COVID-19 patients who received anti-fibrotic therapy obtained a better response in pulmonary function (Kerget et al., 2022). What we do know is that PF is a risk factor for illness severity in a variety of acute and chronic disorders. Meanwhile, COVID-19 patients are prone to have PF sequelae, to find risk factors for PF is particularly important in patients with COVID-19.

Although the relationships between demographic characteristics, clinic symptoms, laboratory findings and comorbidities with PF have been discussed, the mechanism of PF is unclear. Together with previous studies, we surmise the causes to be as follows. The first is the inflammation response. Plenty of studies have showed PF associated with inflammation (Ojo et al., 2020; Zhang et al., 2020; Wendisch et al., 2021). COVID-19 was thought to result in severe inflammation, even inflammatory storm. Consistent with previous researches (Ojo et al., 2020; Li et al., 2021; Hasegawa et al., 2021), in this study, inflammation parameters altered at the early stage after admission, with CRP, ESR, and PCT significantly elevated, as well as Lys significantly decreased. In addition, we discovered that the inflammatory reactions appeared to be more potent in COVID-19 patients with PF that that without PF, making the modifications even more pronounced. What is more, our study found that CRP and ESR were positively correlated with PF from T0 to T4. Secondly, the development of PF may be connected with coagulation/fibrinolysis pathway. The data showed that not only D-dimer, PT, Fib positively correlated with PF ≥ one time point, but also APTT was inversely related. At the same time, the D-dimer demonstrated a significant increasing trend, particularly in COVID-19 with PF.

Additionally, there were other interesting results. We found a significantly negative association of PF with ALB in COVID-19 patients. ALB was significantly related to the outcomes of patients with COVID-19 and depended on many factors (Acharya et al., 2021; Sanson et al., 2022).The deceased levels of ALB may be explained by the persistent inflammatory response, making the production decreased and consumption increased. In addition, our study also found that HGB was negatively correlated with PF, with a distinct decline of the HGB in the COVID-19 patients with PF, this is in line with previous studies, which indicated a lower HGB level at hospital presentation could be a potential predictor for COVID-19 severity (Zhang et al., 2020; Algassim et al., 2021).

We are conscious of the potential limits of our research. On the one hand, in comparison to prospective and interventional research, the time of CT after discharge were different, this study’s retrospective methodology yields less conclusive results. On the other hand, we evaluated pulmonary fibrosis only by CT findings, it would have been much more efficient if we had a diffusion pulmonary function test for the effect of fibrosis (Kerget et al., 2022). In addition, our study does not go into much depth about the risk factors for PF in patients with COVID-19. PF as a sequela of COVID-19 requires long-term follow-up, while our study only analyzed data from a relatively early period, 15 days from admission. Lastly, we only included data from one single center, the results may be more accurate if there is a multicenter study. It has to be illustrated in depth to better identify the risk of PF and intervene in the process of PF, which will need a lot more work.

In conclusion, our study compared clinical data between COVID-19 patients with or without PF and summarized the risk factors for PF. Combining various factors, age was an independent risk factor for PF in patients with COVID-19 and PF was significantly related with clinic parameter of inflammation, coagulopathy and fibrinolysis. Our study showed that PF was positively associated with CRP and ESR, and negatively related to HGB and ALB. Despite certain restrictions, we may draw the conclusion that PF is strongly associated with inflammation/coagulopathy/fibrinolysis. To show specifics of the problem, a lot more effort will be required.

## Data availability statement

The datasets presented in this article are not readily available. Please contact with the corresponding authors. Requests to access the datasets should be directed to YZ, zhongyanjun@csu.edu.cn.

## Ethics statement

The study was approved by the institutional ethics board of the Second Xiangya Hospital of Central South University (No. 2020001). Written informed consent from the participants’ legal guardian/next of kin was not required to participate in this study in accordance with the national legislation and the institutional requirements. Written informed consent was not obtained from the individual(s), nor the minor(s)’ legal guardian/next of kin, for the publication of any potentially identifiable images or data included in this article.

## Author contributions

FL, FLL, and YZ were involved in study design, interpreting data, statistical analysis, and writing of the manuscript. JD, YS, CW, BY, GW, and JL were involved in collecting data. All authors contributed to the article and approved the submitted version.
